# Complex behavior in a modified Jansen and Rit neural mass model

**DOI:** 10.1186/1471-2202-12-S1-P5

**Published:** 2011-07-18

**Authors:** Andreas Spiegler, Thomas R Knösche, Jens Haueisen, Fatihcan M Atay

**Affiliations:** 1 Max Planck Institute for Human Cognitive and Brain Sciences, Leipzig, 04103, Germany; 2 Institute for Biomedical Engineering and Informatics, Ilmenau University of Technology, Ilmenau, 98684, Germany; 3 Max Planck Institute for Mathematics in the Sciences, Leipzig, 04103, Germany

## 

Neural mass models (NMM) explain dynamics of neuronal populations and were designed to strike a balance between mathematical simplicity and biological plausibility [1]. It has been demonstrated that, even in the absence of any time-variant input, they are capable of producing a number of biologically relevant behavior [1]. However, cortical input is often periodic, since neural ensembles tend to oscillate intrinsically or due to rhythmic external stimuli [2]. Here, we investigate the Jansen and Rit NMM for a cortical area [1], comprising three neural masses for pyramidal cells and inhibitory and excitatory interneurons, in response to periodic stimulus of varying frequency.

We consider periodic pulse-like input and systematically vary the normalized input frequency between >0 and 18.5·10^–2^ around the intrinsic frequency (10.8·10^–2^) of the unperturbed NMM (arising from Andronov-Hopf bifurcations) [1]. The normalized stimulus amplitude (*ζ* = 1.5) is located within the effective extrinsic input range [1]. The parameter space is charted by means of Lyapunov spectra, Kaplan-Yorke dimension, time series and power spectra.

**Figure 1:**
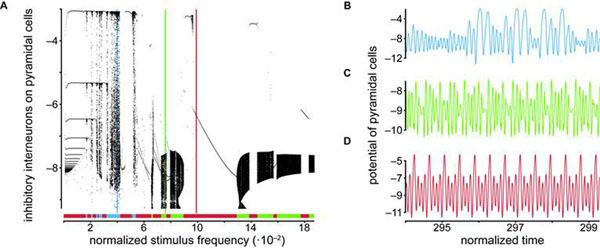
Complex behavior of a periodically forced neural mass model. A Poincaré map against stimulus frequency **A** and three exemplary time series (vertical lines in **A**) for chaotic **B**, quasiperiodic **C** and periodic behavior **D** are shown. The behavior ranges are color coded and indicated by the horizontal line in **A**.

We find complex behavior like entrainment, chaos, and periodic and quasi-periodic motion for biologically plausible parameter ranges without considering noise processes (see Figure 1). Rhythmic and chaotic brain states are found virtually next to each other, such that small parameter changes can give rise to switching from one to another.

We conclude that a periodically forced Jansen and Rit NMM can yield very complex dynamics, including chaos, for plausible parameters. Such complex behavior could explain multi-stability in M/EEG data, which can be observed, for instance, in perception (e.g., binocular rivalry), stages of sleep, changes in attention or vigilance, progression of diseases (e.g., epilepsy), and effects of medication. As an example, we have shown that this model reproduces the resonance phenomena in a clinically relevant photic driving experiment [2].
